# Red blood cell prescription and recognition of transfusion reactions by pediatricians

**DOI:** 10.31744/einstein_journal/2020AO5446

**Published:** 2020-09-09

**Authors:** Carlos João Schaffhausser, João Carlos Pina Faria, Fabíola Isabel Suano-Souza, Roseli Oselka Saccardo Sarni

**Affiliations:** 1 Universidade Municipal de São Caetano do Sul São Caetano do SulSP Brazil Universidade Municipal de São Caetano do Sul, São Caetano do Sul, SP, Brazil.; 2 Centro Universitário Saúde ABC Santo AndréSP Brazil Centro Universitário Saúde ABC, Santo André, SP, Brazil.

**Keywords:** Transfusion medicine, Child, Erythrocyte transfusion, Transfusion reaction, Education, medical

## Abstract

**Objective:**

To assess the level of knowledge of emergency pediatricians on red blood cell transfusions and their reactions.

**Methods:**

Written survey with emergency pediatricians from a pediatric hospital.

**Results:**

Less than 20% of pediatricians showed appropriate knowledge on prescribing red blood cells and recognition of transfusion reactions. There was no significant statistical regarding time since graduation and blood transfusion classes in undergraduate studies or during medical residency.

**Conclusion:**

Pediatricians have insufficient knowledge about red blood cell transfusions and recognition of transfusion reactions.

## INTRODUCTION

Transfusion of red blood cell (RBC) is not exempt from risks. Several transfusion reactions can occur, some of which are fatal.^(1)^ Severe anemia in sick children is a cause of increased mortality, and RBC transfusion can improve survival.^(2)^ On the other hand, unnecessary transfusions of RBC also increase mortality of hospitalized children.^(3)^

Children belong to a group at higher risk of receiving unnecessary blood transfusion.^(4)^ Unit prescription, instead of calculating the volume per body weight (kilogram), leads to greater risk of volume overload, which is related to worse outcomes in children.^(5)^ Circulatory overload is one of the three major causes of transfusion-related deaths.^(6)^ Another important decision is choosing the RBC subtype. Prescribing the correct RBC subtype (filtered, irradiated, washed or phenotyped) contributes to reducing transfusion reactions.^(1)^

The transfusion trigger in asymptomatic children is 7.0g/dL. When anemia is symptomatic or associated with chronic conditions, such as cyanogenic heart disease, lung diseases, etc., the trigger is higher.^(1)^ Sepsis is the major cause of transfusion in symptomatic children in our region and the trigger is 10.0g/dL.^(7)^ The volume of RBC prescribed should be between 10 and 15mL/kg.^(1)^ The main indications for specific RBC subtypes are filtered (immunosuppressed and polytransfused children), irradiated (immunosuppressed and related donors), washed (previous transfusion-related anaphylaxis), and phenotyped (polytransfused patients).^(1)^

Besides immediate transfusion reactions, late reactions can also occur. One of the medium and long-term effects secondary to blood transfusion is abnormal immune response, resulting in increased risk of some types of cancer.^(8)^ Another transfusion risk is related to transmission of new infectious agents, such as the Zika virus, and serology tests are no done routinely.^(9)^ Hence, the decision to transfuse a patient must be cautious.

Although restrictive transfusion (when hemoglobin is below 7.0g/dL) is recommended, many physicians, unaware of the protocols, carry out liberal transfusion (transfusion with higher hemoglobin values).^(10)^ Since this is an important procedure conducted on critically ill children, blood transfusion should be better discussed at undergraduate and graduate levels. Educational measures are proven to improve physicians’ knowledge on transfusion.^(11)^

## OBJECTIVE

To assess the knowledge of pediatric urgency and emergency physicians of a pediatric hospital on prescription of red blood cells and recognition of transfusion reactions.

## METHODS

This is a cross-sectional study of the *Centro Universitário de Saúde ABC, Faculdade de Medicina ABC*, in Santo André (SP, Brazil), carried out by completion of a standardized form, which was applied to pediatricians at an urgency and emergency department of *Hospital Infantil e Maternidade Márcia Braido*, in São Caetano do Sul (SP).

The hospital provides care via the National Health System (SUS – *Sistema Único de Saúde*) and health insurance companies, with an average of 9,000 visits per month. The pediatric emergency department has an emergency room with 3 beds; observation room with 9 beds; 6 fast-track rooms; an 18-bed ward; and a 5-bed pediatric intensive care unit.

The study period was from February to March 2018. All 74 pediatricians working at the pediatric emergency department were invited to participate in the study.

The form was delivered printed, with no request for participant identification, accompanied by an Informed Consent Form (ICF). Participants had 30 minutes to read and answer the questions. The form had nine objective questions based on frequent clinical situations, participants were asked to indicate the correct alternative; there was also a list of possible signs/symptoms for participants identifying those they believed to be transfusion reactions (Annex 1).

This form was prepared after discussing with blood transfusion specialists and pediatricians who work at emergency hospitals and pediatric emergency departments. The most relevant topics were addressed, taking into account their importance to score each question. In a pilot study using the validated version, there was low adherence to the questionnaire; therefore a new form was created. It was concise and for quick completion, aiming to have greater participation in the study.

The score was prepared as follows: questions 1 to 3 of the form covered transfusion trigger and volume calculation; therefore, they were more relevant and 2 points were assigned for each correct answer. The remaining questions received 1 point for each correct answer. The score on the indications could vary between zero and 12. The minimum score considered as adequate was 8 points. Regarding the recognition of transfusion reactions, each properly recognized reaction received 1 point (ranging from zero to 15 points). The minimum score necessary to consider knowledge as adequate was 10 points. The signs and symptoms unrelated to transfusion reactions that were recognized as such, received 1 point each, except for the question on 0.5^o^C rise in temperature, which received 2 points when correctly marked (ranging from zero to 8 points). Fever is one of the most frequent transfusion reactions and, when it occurs, the transfusion must be interrupted. The inaccurate diagnosis of transfusion-related fever results in inappropriate management, justifying the higher score for this question. The minimum score necessary to consider knowledge adequate was 5 points.

In order to determine the appropriate responses, we considered the current recommendations of the Ministry of Health.^(1)^ When the topic was not addressed by the Ministry of Health, we used recommendations from the United States.^(12,13)^ The transfusion trigger is considered adequate when hemoglobin is <7.0g/dL. The indication for children in septic shock is hemoglobin <10.0g/dL.^(12,13)^ The adequate volume is between 10 and 15mL/kg.^(1)^ The transfusion should last between one and 4 hours, in order to avoid circulatory overload and hypothermia in a shorter interval, and bacterial contamination of the blood product in a longer interval.^(1)^ The specific indication of subtypes is only for especial situations, such as filtered RBC for polytransfused patients or to prevent transmission of cytomegalovirus in immunocompromised patients; irradiated RBC to avoid transfusion-associated graft *versus* host disease in immunocompromised patients or related donation; washed RBC to remove plasma proteins that cause anaphylaxis; phenotyped RBC to prevent erythrocyte alloimmunization in polytransfused patients; and warm RBC to prevent hypothermia in massive transfusion for trauma.^(1)^

Incomplete forms or with more than one alternative checked in the objective questions were excluded.

The study was approved by the Internal Review Board of the *Fundação Municipal de Saúde de São Caetano do Sul* (FUMUSA), opinion 1.783.673, CAAE: 56596216.7.0000.5635.

The database was built using the Excel software. For statistical analysis, Epi Info^TM^, version 7.2.2.6, was used. Continuous variables were tested for normality, using the Shapiro-Wilk test; those with parametric distribution were presented as means (standard deviation); and the non-parametric variables, as median (minimum and maximum).

Qualitative variables were presented as absolute number and percentages, and were compared using the χ^2^ test or Fisher’s exact test. Significance level was set at 5%.

## RESULTS

A total of 67 pediatricians completed the form (90.5%). The median time since graduation was 7 years (1 to 44 years); 61 (91%) professionals had prescribed transfusion of blood components.

In the sample, 43 (64%) pediatricians had classes on blood transfusion during undergraduate medical course and, of these, 83.7% had less than 2 hours/class. In total, 86.6% considered this time insufficient. Among respondents, 18 (26.9%) had a class on blood transfusion during medical residency in pediatrics, and 72.2% had less than 2 hours/class. A total of 98.5% of participants considered this time insufficient.

Sixty-two (92.5%) professionals were willing to participate in a continuous education program on blood transfusion.

When asked about the transfusion trigger (hemoglobin value) to indicate RBC to a child with acute anemia, with no underlying disease, and no signs of hemodynamic decompensation, 41.8% checked the appropriate answer (7g/dL). The transfusion trigger in children with septic shock was correctly marked by 28.4% (10g/dL).

Adequate RBC volume was indicated by 28 (35.8%) pediatricians. The adequate RBC infusion time in children with no hemodynamic decompensation was correctly indicated by 46.3% (1 to 4 hours).

Filtered red blood cells were correctly indicated by 28.4% (hemoglobinopathies) of sample; irradiated by 52.2% (severe immunodeficiency); washed by 25.4% (previous transfusion-related anaphylaxis); phenotyped by 34.3% (hemoglobinopathies); and warmed by 68.7% (polytrauma with massive transfusion).

There was no difference between the groups when time since graduation (p=0.447) and having received a class during undergraduate medical course (p=0.407) or medical residency (p=1) were compared regarding adequacy of responses to the questionnaire (Table 1).

**Table 1:** Pediatricians’ knowledge on red blood cell prescription regarding time since graduation and previous classes on blood transfusion in undergraduate medical course or residency

Variables	Knowledge		p value
	Inadequate n (%)	Adequate n (%)	
Time since graduation, years			
Up to 7	28 (93.3)	2 (6.7)	0.447
7 or more	32 (86.5)	5 (13.5)	
Had class in undergraduate medical course			
Yes	37 (86)	6 (14)	0.407
No	23 (95.8)	1 (4.2)	
Had class in residency			
Yes	16 (88.9)	2 (11.1)	1
No	44 (89.8)	5 (10.2)	

Responses on signs and symptoms related to transfusion reactions, change in urine color, jaundice, and hypoxia, were more than 80% inadequate. Regarding the signs and symptoms not related to reactions, a rise in temperature by 0.5^°^C had 41% inadequacy (Table 2). There was no difference between the groups when time since graduation and having classes during undergraduate medical course or medical residency were compared regarding influence in recognition of signs and symptoms related or not to transfusion reactions (Table 3).

**Table 2:** Signs and symptoms related and unrelated to transfusion reactions

Sign/symptom	Correct n (%)	Incorrect n (%)
Signs and symptoms related to transfusion reactions		
Change in urine color	6 (9)	61 (91)
Jaundice	10 (14.9)	57 (85.1)
Hypoxia	13 (19.4)	54 (80.6)
Hypotension	15 (22.4)	52 (77.6)
Hypertension	17 (25.4)	50 (74.6)
Chest pain	19 (28.4)	48 (71.6)
Pain at infusion site	23 (34.3)	44 (65.7)
Abdominal pain	26 (38.8)	41 (61.2)
Chills	32 (47.8)	35 (52.2)
Nausea	35 (52.2)	32 (47.8)
Shock	42 (62.7)	25 (37.3)
Dyspnea	45 (67.2)	22 (32.8)
Rise in temperature >1ºC	46 (68.7)	21 (31.3)
Edema	50 (74.6)	17 (25.4)
Urticaria	61 (91)	6 (9)
Signs and symptoms not related to transfusion reactions		
Rise in temperature by 0.5ºC	39 (58.2)	28 (41.8)
Diarrhea	49 (73.1)	18 (26.9)
Odynophagia	55 (82)	12 (18)
Toothache	57 (85)	10 (15)
Dysuria	62 (92.5)	5 (7.5)
Otalgia	62 (92.5)	5 (7.5)
Alopecia	64 (95.5)	3 (4.5)

**Table 3:** Knowledge on transfusion reactions related to red blood cell transfusion

Variables	Knowledge		p value
	Inadequate n (%)	Adequate n (%)	
Signs and symptoms related to transfusion reaction			
Time since graduation, years			
Up to 7	27 (90)	3 (10)	1
7 or more	34 (91.9)	3 (8.1)	
Had class in undergraduate medical course			
Yes	37 (86)	6 (14)	0.080
No	24 (100)	0 (0)	
Had class in residency			
Yes	15 (83.3)	3 (16.7)	0.331
No	46 (93.9)	3 (6.1)	
Signs and symptoms not related to transfusion reactions			
Time since graduation, years			
Up to 7	7 (23.3)	23 (76.7)	0.349
7 or more	5 (13.5)	32 (86.5)	
Had class in undergraduate medical course			
Yes	7 (16.3)	36 (83.7)	0.743
No	5 (20.8)	19 (71.2)	
Had class in residency			
Yes	1 (5.6)	17 (94.4)	0.158
No	11 (22.4)	38 (77.6)	

## DISCUSSION

The study had good adherence of pediatricians from the urgency and emergency department of the hospital evaluated. The age of the participants varied widely with an interquartile range of 15.5 years; 9 out of 10 had prescribed RBC previously.

Although two thirds of participants had classes on blood transfusion during undergraduate medical course, the majority reported that class hour load was not sufficient. A quarter of the participants had classes during medical residency, and almost all reported time was not enough either. A study carried out in Oman with 130 residents found that 49% of physicians said it was necessary to increase the class hour load during the last year of undergraduate course, and 94.5% at the medical residency.^(14)^ In our study, most pediatricians had less than 2 hours/class during undergraduate course or residency. A multicenter study carried out in nine countries described that 53% and 55% had less than 2 hours/class in undergraduate course and in residency, respectively.^(15)^ These data demonstrate pediatricians at this hospital had a class hour load in blood transfusion smaller than the average found in organizations abroad.

The responses for RBC transfusion trigger were mostly inadequate, mainly in the case of septic shock. In this situation, the trigger in pediatric patients is 10g/dL.^(16)^ The volume was appropriately described by one third of pediatricians. Among the participants, 61.2% indicated volumes higher than ideal. The prescription of excessive volumes increases the risk of circulatory overload, a transfusion reaction that presents high mortality (12%).^(6)^ The correct indication of RBC subtypes varied between 25.4% and 68.7%. Prescribing the correct subtypes increases transfusion yield and decreases transfusion reactions.^(1)^ Prescribing the correct subtypes contributes to reducing hospital treatment costs.^(17)^

Different studies using other questionnaire models found appropriate answers about RBC transfusion between 32% and 56%.^(15,18)^ A retrospective study carried out in the same region of the study, with 837 RBC transfusions in children, demonstrated adequate indication in 65.3% and correct volume in 58.8%.^(19)^

Time since graduation was not relevant to adequate answers, and this was corroborated by a similar study.^(19)^

Regarding the recognition of signs and symptoms of transfusion reactions, more than 70% did not recognize the following as suggestive of these reactions: change in urine color, jaundice, hypoxia, hypotension, hypertension, and chest pain. A Brazilian study identified that 3.8% of children had transfusion reactions.^(20)^ This percentage is equivalent to that seen in other countries.^(21)^ Transfusion reactions increase mortality, especially when there is delay in recognizing them.^(22)^ The non-hemolytic febrile reaction is one of the most prevalent, being defined by a ≥1^o^C rise in temperature.^(1)^ Just over half respondents managed to recognize this minimum temperature rise as transfusion reaction.

The data found demonstrated time since graduation was not related to greater knowledge of good transfusion practices, suggesting that actions on continuing education are necessary.

Most participants stated that they would like to engage in continuing education in transfusion medicine. Continuing education has proven to enhance knowledge of physicians and other health professionals about blood transfusion.^(10)^ All physicians who prescribe blood components should receive training in transfusion medicine - including the pediatric emergency medicine physicians.^(23)^

The fact that having a class or not does not influence in adequate knowledge on blood transfusion suggests that the applied teaching methodology is not suitable for the subject. This has already been proven in a study carried out in the United Kingdom.^(23)^ Studies indicated that exposing the topic in different ways to undergraduate students and residents enhnaces knowledge about transfusion medicine.^(24)^ The active teaching methodology seems to be more effective, especially if adapted to the undergraduate course and the specialty of residency.^(25)^ Teaching based on specific skills applied to clinical cases favors student learning.^(26)^ A Brazilian study assessed knowledge of sixth-year undergraduate medical students and residents of several areas. The transfusion adequacy was 35% and 49.5%, respectively.^(27)^ In 2015, 249 Brazilian Medical Schools were evaluated, and only 3.9% of courses covered transfusion medicine.^(28)^ Similar data have been described in other countries.^(29,30)^

As limitations of this study we have memory bias regarding having received information during the undergraduate course and residency on blood transfusion, the selection of emergency physicians, and the fact of being a single center study.

## CONCLUSION

Pediatricians in the urgency and emergency department of this hospital have insufficient knowledge about prescription and recognition of reactions related to red blood cell transfusion in children.

The data found show deficiency in training of physicians and pediatricians on blood transfusion.

A proposal was made for a training program in transfusion medicine to be implemented in the unit.

## Annex 1

**Table:** Form sent to pediatricians of the pediatric emergency and urgency department of *Hospital Infantil e Maternidade Márcia Braido*, in São Caetano do Sul (SP), Brazil

General data									
Time since graduation (in years)			__________ years						
Have you prescribed transfusion?			Yes ( )			No ( )			
Did you have blood transfusion classes in undergraduate medical course?			Yes ( )			No ( )			
Hour-load of blood transfusion classes in undergraduate medical course			0 hour ( )	Up to 2 hours ( )		2 to 4 hours ( )			More than 4 hours ( )
Assessment of hour-load			Adequate ( )		Little ( )			Too much ( )	
Did you have classes on blood transfusion in residency?			Yes ( )			No ( )			
Hour-load of blood transfusion classes in residency?			0 hour ( )	Up to 2 hours ( )		2 to 4 hours ( )			More than 4 hours ( )
Assessment of hour-load			Adequate ( )		Little ( )			Too much ( )	
Would you like to participate in continuous education?			Yes ( )			No ( )			
**Form**									
1. For children with acute anemia with no hemodynamic decompensation, red blood cell transfusion is indicated when hemoglobin (g/dL) level is under:									
a) 6	b) 7	c) 8		d) 9			e) 10		
2. For children with anemia and septic shock, red blood cell transfusion is indicated when hemoglobin (g/dL) level is under:									
a) 6	b) 7	c) 8		d) 9			e) 10		
3. Which volume (mL/kg) of red blood cells should be prescribe to pediatric patients?									
a) Up to 5	b) 5 to 10	c) 10 to 15		d) 15 to 20			e) >20		
4. What is the infusion time (hours) of red blood cells in a child with acute anemia, and no signs of hemodynamic decompensation?									
a) Open	b) Up to 1	c) 1 to 4		d) 4 to 8			e) 8 to 12		
5. In each situation are filtered red blood cells indicated?									
a) Previous anaphylaxis to transfusion	b) Acute kidney failure	c) Heart disease		d) Related donor			e) Hemoglobinopathies		
6. In each situation are irradiated red blood cells indicated?									
a) Severe immunodeficiency	b) Hemoglobinopathies	c) Previous anaphylaxis		d) Dengue infection			e) Lung disease		
7. In each situation are washed red blood cells indicated?									
a) Risk group donor	b) Acute kidney failure	c) Blood marrow transplant		d) Previous anaphylaxis to transfusion			e) Severe immunodeficiency		
8. In each situation are phenotyped red blood cells indicated?									
a) Hemoglobinopathies	b) IgA immune deficiency	c) Heart disease		d) Related donor			e) Newborn		
9. In each situation are warm red blood cells indicated?									
a) Chemotherapy	b) Blood marrow transplant	c) Hemoglobinopathies		d) Polytrauma and massive transfusion			e) Previous anaphylaxis to transfusion		
Tick (X) transfusion reaction-related signs and symptoms:									
( ) Chills									
( ) Axillary temperature rise by 1^°^C or more									
( ) Fever after rise by 0.5^°^C increase									
( ) Alopecia up to 72 hours after the transfusion									
( ) Abdominal pain									
( ) Chest pain									
( ) Pain at the infusion site									
( ) Diarrhea									
( ) Hypertension									
( ) Hypotension									
( ) Toothache									
( ) Dyspnea									
( ) Hypoxia									
( ) Jaundice									
( ) Dysuria									
( ) Urticaria									
( ) Localized or generalized edema									
( ) Otalgia									
( ) Nausea									
( ) Shock									
( ) Changes in urine color									
( ) Odynophagia									
