# Supplementary material for: Red blood cell prescription and recognition of transfusion reactions by pediatricians
Source: Einstein (Sao Paulo). 2020 Sep 9;18:eAO5446. doi: 10.31744/einstein_journal/2020AO5446 (PMC7480493; doi:10.31744/einstein_journal/2020AO5446)
Supplement: Translation

# Translation: Prescrição de hemácias e reconhecimento de reações transfusionais por pediatras

**Authors:** Carlos João Schaffhausser, João Carlos Pina Faria, Fabíola Isabel Suano-Souza, Roseli Oselka Saccardo Sarni

**Affiliations:** 1 Brasil Universidade Municipal de São Caetano do Sul, São Caetano do Sul, SP, Brasil.; 2 Brasil Centro Universitário Saúde ABC, Santo André, SP, Brasil.

**Keywords:** Medicina transfusional, Criança, Transfusão de eritrócitos, Reação transfusional, Educação médica

## Abstract

**Objetivo:**

Avaliar o conhecimento de pediatras emergencistas sobre transfusão de concentrados de hemácias e reações transfusionais.

**Métodos:**

Aplicação de formulário para pediatras emergencistas de um hospital pediátrico.

**Resultados:**

Menos de 20% dos participantes demonstraram conhecimento adequado sobre prescrição de concentrados de hemácias e reconhecimento de reações transfusionais. Não houve diferença estatística significativa quando avaliados o tempo de formação profissional e o fato de ter recebido aula de hemoterapia na graduação ou na residência médica.

**Conclusão:**

Os pediatras têm conhecimento insuficiente sobre prescrição de concentrados de hemácias e reconhecimento de reações transfusionais.

## INTRODUÇÃO

A transfusão de concentrado de hemácias (CH) não é isenta de riscos. Podem ocorrer diversas reações transfusionais, sendo algumas fatais.^(1)^ A anemia grave em crianças doentes é causa de aumento de mortalidade, e a transfusão de CH pode melhorar a sobrevida.^(2)^ Por outro lado, a transfusão desnecessária de CH também aumenta a mortalidade de crianças hospitalizadas.^(3)^

Crianças fazem parte do grupo com maior risco de receber uma transfusão de sangue desnecessária.^(4)^ A prescrição por unidade, no lugar do cálculo do volume por quilograma de peso, resulta em maior risco de sobrecarga de volume, que relaciona-se com pior desfecho em crianças.^(5)^A sobrecarga circulatória é uma das três principais causas de morte relacionadas à transfusão.^(6)^ Outra decisão importante é a escolha do subtipo de CH. A prescrição do subtipo correto de CH (filtrado, irradiado, lavado e fenotipado) contribui para reduzir as reações transfusionais.^(1)^

O gatilho transfusional em crianças assintomáticas é de 7,0g/dL. Quando a anemia é sintomática ou associada à doença crônica, como cardiopatia cianogênica, pneumopatias etc., o gatilho é superior a esse valor.^(1)^ A sepse é a maior causa de transfusão em crianças sintomáticas em nossa região e possui gatilho de 10,0g/dL.^(7)^ O volume prescrito de CH deve estar entre 10 e 15mL/kg.^(1)^As principais indicações de subtipos específicos de CH são filtrados (imunodeprimidos e politransfundidos), irradiados (imunodeprimidos e doação aparentada), lavados (anafilaxia prévia relacionada à transfusão) e fenotipados (politransfundidos).^(1)^

Além de reações transfusionais imediatas, algumas tardias também podem ocorrer. Um dos efeitos a médio e longo prazo secundários à transfusão sanguínea é a modificação na resposta imune, resultando em aumento do risco de alguns tipos de câncer.^(8)^ Outro risco transfusional está relacionado à transmissão de novos agentes infecciosos, como o Zika vírus, para os quais não são realizadas sorologias na rotina.^(9)^ Por esse motivo, a decisão de se transfundir um paciente deve ser criteriosa.

Apesar da transfusão restritiva (transfundir quando a hemoglobina estiver abaixo de 7,0g/dL) ser recomendável, muitos médicos exercem a transfusão liberal (transfusão com valores maiores de hemoglobina) por desconhecimento dos protocolos.^(10)^ Por tratar-se de procedimento importante realizado em crianças gravemente doentes, a hemoterapia deveria ser melhor discutida na graduação e na pós-graduação. Medidas educativas melhoram comprovadamente o conhecimento de médicos sobre transfusão.^(11)^

## OBJETIVO

Avaliar o conhecimento de médicos pediatras do setor de urgências e emergências de um hospital pediátrico sobre prescrição de concentrado de hemácias e reconhecimento de reações transfusionais.

## MÉTODOS

Trata-se de um estudo transversal do Centro Universitário de Saúde ABC, Faculdade de Medicina ABC, em Santo André (SP), realizado por meio de formulário padronizado, que foi aplicado aos médicos pediatras da unidade de urgências e emergências do Hospital Infantil e Maternidade Márcia Braido, em São Caetano do Sul (SP).

O hospital atende pelo Sistema Único de Saúde (SUS) e convênios, com média de 9.000 atendimentos ao mês. O pronto-socorro infantil possui sala de emergência com 3 leitos; sala de observação com 9 leitos; 6 salas de pronto atendimento; enfermaria com 18 leitos; e unidade de terapia intensiva pediátrica com 5 leitos.

O período do estudo foi de fevereiro a março de 2018. Todos os 74 pediatras do pronto-socorro infantil foram convidados a participar do estudo.

O formulário foi entregue impresso em papel, sem solicitação de identificação do participante da pesquisa, acompanhado do Termo de Consentimento Livre e Esclarecido (TCLE). O participante teve 30 minutos para ler e responder as questões. Esse formulário possuía nove questões objetivas baseadas em situações clínicas frequentes, sendo solicitado assinalar a alternativa correta e uma lista de possíveis sinais/sintomas para o participante identificar os que acreditava ser reações transfusionais (Anexo 1).

Esse formulário foi elaborado após discussão com especialistas em hemoterapia e pediatras, que trabalham em hospitais de urgências e emergências pediátricas. Foram abordados os tópicos mais relevantes, levando-se em conta sua importância para a pontuação de cada questão. Optou-se por criar um formulário novo, pois, em um estudo piloto com a utilização da versão validada, houve baixa adesão ao questionário. O formulário foi redigido de forma concisa e para rápido preenchimento, com o objetivo de ter maior participação no estudo.

O escore foi elaborado da seguinte maneira: as questões 1 a 3 do formulário abordaram gatilho transfusional e cálculo de volume e, por isso, apresentavam maior relevância, sendo atribuídos 2 pontos para cada acerto. As demais questões receberam 1 ponto para cada acerto. Assim, a pontuação sobre as indicações podia variar entre zero e 12. A pontuação mínima considerada para definir como adequado o conhecimento do pediatra era de 8 pontos. Em relação ao reconhecimento das reações transfusionais, cada reação reconhecida adequadamente recebeu 1 ponto (variando de zero a 15 pontos). A pontuação mínima necessária para considerar o conhecimento adequado era de 10 pontos. Os sinais e sintomas não relacionados com reações transfusionais que foram reconhecidos dessa forma receberam 1 ponto cada, exceto a questão envolvendo a elevação de 0,5^o^C na temperatura do paciente, que recebeu 2 pontos quando assinalada corretamente (variando de zero a 8 pontos). A febre é uma das reações transfusionais mais frequentes e, quando ocorre, a transfusão deve ser interrompida. O diagnóstico impreciso de febre relacionada à transfusão resulta em condutas inadequadas, justificando a maior pontuação para essa questão. A pontuação mínima necessária para considerar o conhecimento adequado era de 5 pontos.

Para a consideração das respostas adequadas, consideramos as recomendações atuais do Ministério da Saúde.^(1)^ Quando o tema não era abordado pelo Ministério da Saúde, utilizamos as recomendações americanas.^(12,13)^ O gatilho transfusional é considerado adequado quando a hemoglobina do paciente encontra-se <7,0g/dL. A indicação para crianças em choque séptico é hemoglobina <10,0g/dL.^(12,13)^ O volume adequado está entre 10 e 15mL/kg.^(1)^ A transfusão deve ser realizada entre 1 e 4 horas, para evitar sobrecarga circulatória e hipotermia em intervalo menor, e contaminação bacteriana do hemocomponente em intervalo maior.^(1)^ A indicação específica de subtipos é realizada apenas em situações específicas, como CH filtrado para pacientes politransfundidos e para evitar transmissão de citomegalovírus em imunocomprometidos; CH irradiados para evitar a reação do enxerto versus hospedeiro transfusional em imunocomprometidos e doação aparentada; CH lavados para retirar proteínas plasmáticas causadoras de anafilaxia; CH fenotipadas para evitar aloimunização eritrocitária em politransfundidos e CH aquecido para evitar hipotermia na transfusão maciça do trauma.^(1)^

Foram excluídos formulários incompletos ou com mais de uma alternativa assinalada nas questões objetivas.

O estudo foi aprovado pelo Comitê de Ética em Pesquisa da Fundação Municipal de Saúde de São Caetano do Sul (FUMUSA), parecer 1.783.673, CAAE: 56596216.7.0000.5635.

O banco de dados foi construído com o uso do programa Excel. Para a análise estatística, utilizou-se o Epi Info^TM^ ,versão 7.2.2.6. As variáveis contínuas foram testadas quanto à sua normalidade, por meio do teste de Shapiro-Wilk; as que apresentavam distribuição paramétrica foram apresentadas na forma de média (desvio padrão); e as que eram não paramétricas mediana (mínimo e máximo). As variáveis qualitativas foram apresentadas na forma de número absoluto e percentual, tendo sido comparadas por meio do teste χ^2^ ou teste exato de Fisher. O nível de significância adotado foi de 5%.

## RESULTADOS

Responderam ao formulário 67 pediatras (90,5%). A mediana de tempo de formação dos participantes foi de 7 anos (1 a 44 anos); 61 (91%) profissionais já tinham prescrito transfusão de hemocomponentes.

Na amostra, 43 (64%) pediatras tiveram aula sobre hemoterapia na graduação e, destes, 83,7% tiveram menos de 2 horas/aula. No total, 86,6% consideraram esse tempo insuficiente. Dentre os respondentes, 18 (26,9%) tiveram aula sobre hemoterapia na residência médica de pediatria, e 72,2% tiveram menos de 2 horas/aula. No total, 98,5% consideraram esse tempo insuficiente.

Desejavam participar de um programa de educação permanente sobre hemoterapia 62 (92,5%) profissionais.

Quando questionados sobre o gatilho transfusional (valor da hemoglobina) para indicar CH em criança com anemia aguda, sem doença de base e sem sinais de descompensação hemodinâmica, 41,8% assinalaram a resposta adequada (7g/dL). O gatilho transfusional em crianças com choque séptico foi assinalado corretamente por 28,4% (10g/dL).

O volume adequado de CH foi indicado por 28 (35,8%) pediatras. O tempo adequado de infusão do CH em crianças sem descompensação hemodinâmica foi assinalado corretamente por 46,3% (1 a 4 horas) deles.

Concentrado de hemácias filtradas foi indicado corretamente por 28,4% (hemoglobinopatias) da amostra; irradiadas por 52,2% (imunodeficiência grave); lavadas por 25,4% (anafilaxia prévia relacionada à transfusão anterior); fenotipadas por 34,3% (hemoglobinopatias); e aquecidas por 68,7% (politrauma com transfusão maciça).

Quando foi avaliado se o tempo de formação (p=0,447) e o fato de ter recebido aula na graduação (p=0,407) ou residência médica (p=1) influenciaram na adequação das respostas ao questionário, não encontramos diferença entre os grupos (Tabela 1).

**Tabela 1:** Conhecimento dos pediatras sobre prescrição de concentrado de hemácias em relação ao tempo de formação e ao histórico de aulas de hemoterapia na graduação e na residência médica

Variáveis	Conhecimento		Valor de p
	Inadequado n (%)	Adequado n (%)	
Tempo de formação, anos			
Até 7	28 (93,3)	2 (6,7)	0,447
7 ou mais	32 (86,5)	5 (13,5)	
Teve aula na graduação			
Sim	37 (86)	6 (14)	0,407
Não	23 (95,8)	1 (4,2)	
Teve aula na residência			
Sim	16 (88,9)	2 (11,1)	1
Não	44 (89,8)	5 (10,2)	

Respostas sobre os sinais e sintomas relacionados a reações transfusionais, alteração da cor da urina, icterícia e hipóxia tiveram inadequação superior a 80%. Em relação aos sinais e sintomas não relacionados às reações, o aumento de temperatura de 0,5^o^C apresentou inadequação de 41% (Tabela 2). Quando foi avaliado se o tempo de formação e o fato de ter recebido aula na graduação ou residência médica influenciaram no reconhecimento de sinais e sintomas relacionados ou não a reações transfusionais, não encontramos diferença entre os grupos (Tabela 3).

**Tabela 2:** Sinais e sintomas relacionados e não relacionados a reações transfusionais

Sinal/sintoma	Correto n (%)	Incorreto n (%)
Sinais e sintomas relacionados a reações transfusionais		
Alteração da cor da urina	6 (9)	61 (91)
Icterícia	10 (14,9)	57 (85,1)
Hipóxia	13 (19,4)	54 (80,6)
Hipotensão	15 (22,4)	52 (77,6)
Hipertensão	17 (25,4)	50 (74,6)
Dor torácica	19 (28,4)	48 (71,6)
Dor no local de infusão	23 (34,3)	44 (65,7)
Dor abdominal	26 (38,8)	41 (61,2)
Calafrios	32 (47,8)	35 (52,2)
Náuseas	35 (52,2)	32 (47,8)
Choque	42 (62,7)	25 (37,3)
Dispneia	45 (67,2)	22 (32,8)
Aumento de temperatura >1ºC	46 (68,7)	21 (31,3)
Edema	50 (74,6)	17 (25,4)
Urticária	61 (91)	6 (9)
Sinais e sintomas não relacionados a reações transfusionais		
Aumento de 0,5ºC	39 (58,2)	28 (41,8)
Diarreia	49 (73,1)	18 (26,9)
Odinofagia	55 (82)	12 (18)
Dor de dente	57 (85)	10 (15)
Disúria	62 (92,5)	5 (7,5)
Otalgia	62 (92,5)	5 (7,5)
Alopecia	64 (95,5)	3 (4,5)

**Tabela 3:** Conhecimento das reações transfusionais relacionadas à transfusão de concentrado de hemácias

Variáveis	Conhecimento		Valor de p
	Inadequado n (%)	Adequado n (%)	
Sinais e sintomas relacionados à reação transfusional			
Tempo de formação, anos			
Até 7	27 (90)	3 (10)	1
7 ou mais	34 (91,9)	3 (8,1)	
Teve aula na graduação			
Sim	37 (86)	6 (14)	0,080
Não	24 (100)	0 (0)	
Teve aula na residência			
Sim	15 (83,3)	3 (16,7)	0,331
Não	46 (93,9)	3 (6,1)	
Sinais e sintomas não relacionados à reação transfusional			
Tempo de formação, anos			
Até 7	7 (23.3)	23 (76.7)	0.349
7 ou mais	5 (13.5)	32 (86.5)	
Teve aula na graduação			
Sim	7 (16,3)	36 (83,7)	0,743
Não	5 (20,8)	19 (71,2)	
Teve aula na residência			
Sim	1 (5,6)	17 (94,4)	0,158
Não	11 (22,4)	38 (77,6)	

## DISCUSSÃO

O estudo teve boa adesão dos pediatras do setor de urgências e emergências do hospital avaliado. A idade dos participantes apresentou grande variação com intervalo interquartílica de 15,5 anos, e 9 em cada 10 deles já tinham prescrito CH.

Apesar de dois terços dos participantes terem tido aula de hemoterapia na graduação, a maioria referiu que o tempo foi insuficiente. Um quarto dos participantes teve aula na residência médica, e quase todos referiram que o tempo também foi insuficiente. Estudo realizado em Omã com 130 residentes identificou que 49% dos médicos disseram ser necessário aumentar o tempo do ensino no internato e 94,5% na residência médica.^(14)^ Em nosso estudo, a maioria dos pediatras teve menos de 2 horas/aula na graduação e na residência. Estudo multicêntrico realizado em nove países descreveu que 53% e 55% tiveram menos de 2 horas/aula na graduação e na residência, respectivamente.^(15)^ Esses dados demonstram que os pediatras deste hospital tiveram carga horária de ensino em hemoterapia inferior à média encontrada em instituições de outros países.

O gatilho transfusional de CH teve baixa adequação, principalmente na situação de choque séptico. O gatilho, nessa situação, em pacientes pediátricos, é de 10g/dL.^(16)^ O volume foi descrito adequadamente por um terço dos pediatras. Dentre os participantes, 61,2% indicaram volumes maiores que o ideal. A prescrição de volumes excessivos eleva o risco de sobrecarga circulatória, reação transfusional que apresenta mortalidade elevada (12%).^(6)^ A indicação correta de subtipos de CH variou entre 25,4% e 68,7%. A prescrição do subtipo correto eleva o rendimento transfusional e diminui reações transfusionais.^(1)^A prescrição de subtipos corretos colabora com a redução nos custos do tratamento hospitalar.^(17)^

Estudos diferentes, utilizando outros modelos de questionários, encontraram adequação nas respostas sobre transfusão de CH entre 32% e 56%.^(15,18)^ Levantamento retrospectivo realizado na mesma região do estudo, com 837 transfusões de CH em crianças, demonstrou adequação na indicação de 65,3% e no volume de 58,8%.^(19)^

O tempo de formação profissional não teve relevância nas adequações das respostas, dado semelhante ao observado em estudo similar.^(19)^

Em relação ao reconhecimento de sinais e sintomas de reações transfusionais, mais de 70% não reconheceram os seguintes como sugestivos destas reações: alteração da cor da urina, icterícia, hipóxia, hipotensão, hipertensão e dor torácica. Estudo brasileiro identificou que 3,8% das crianças apresentaram reações transfusionais.^(20)^ Este percentual é equivalente ao observado em outros países.^(21)^As reações transfusionais elevam a mortalidade dos pacientes, especialmente quando ocorre atraso no reconhecimento dessa reação.^(22)^A reação febril não hemolítica é uma das mais prevalentes, sendo definida pela elevação ≥1ºC.^(1)^ Pouco mais da metade conseguiu reconhecer este valor mínimo de elevação da temperatura para classificar como reação transfusional.

Os dados encontrados demonstram que o tempo de formação não teve relação com maior conhecimento das boas práticas transfusionais, sugerindo que medidas de educação permanente são necessárias.

A maioria dos participantes afirmou que gostaria de participar do ensino continuado em hemoterapia. A educação permanente comprovadamente melhora o conhecimento do médico e de outros profissionais da saúde sobre transfusão sanguínea.^(10)^ Todos os médicos que prescrevem hemocomponentes devem receber treinamento sobre medicina transfusional − entre eles o pediatra emergencista.^(23)^

O fato de ter ou não aula não influenciar no conhecimento adequado de hemoterapia sugere que a metodologia de ensino aplicada não é adequada para o assunto. Isso já foi comprovado em estudo realizado no Reino Unido.^(23)^ Estudos indicam que expor o tema de diferentes maneiras a alunos e residentes melhora o conhecimento sobre a hemoterapia.^(24)^ A metodologia ativa de ensino parece ser mais eficaz, principalmente se adaptada ao momento da graduação e da especialidade do médico residente.^(25)^O ensino baseado em competências específicas aplicadas em casos clínicos favorece o aprendizado do aluno.^(26)^Estudo brasileiro avaliou o conhecimento de alunos do sexto ano do curso de medicina e residentes de diversas áreas. A adequação transfusional foi de 35% e 49,5%, respectivamente.^(27)^Em 2015, 249 escolas médicas brasileiras foram avaliadas, e apenas 3,9% dos cursos abordavam medicina transfusional.^(28)^ Dados semelhantes foram descritos em outros países.^(29,30)^

Podem ser descritos como limitações desse estudo o viés de memória em relação a ter recebido informações na graduação e residência sobre hemoterapia, a seleção de uma amostra de profissionais da emergência e o fato de ter sido realizado em centro único.

## CONCLUSÃO

Os pediatras da urgência e emergência deste hospital apresentam conhecimento insuficiente sobre prescrição e reconhecimento sobre reações relacionadas à transfusão de concentrado de hemácias em crianças.

Os dados encontrados demonstram deficiência na formação do médico e pediatras sobre hemoterapia.

Foi apresentada proposta de um programa de capacitação em medicina transfusional para ser implantado na unidade.

## Anexo 1

**Table:** Formulário aplicado aos pediatras do setor de urgências e emergências pediátricas do Hospital Infantil e Maternidade Márcia Braido, em São Caetano do Sul (SP)

Dados gerais									
Tempo de formação (em anos)			__________ anos						
Realizou transfusão?			Sim ( )			Não ( )			
Teve aula de hemoterapia na graduação?			Sim ( )			Não ( )			
Hora/aula de hemoterapia na graduação?			0 hora ( )	Até 2 horas ( )		2 a 4 horas ( )			Mais de 4 horas ( )
Avaliação do tempo de hora/aula			Adequado ( )		Pouco ( )			Muito ( )	
Teve aula de hemoterapia na residência?			Sim ( )			Não ( )			
Hora/aula de hemoterapia na residência?			0 hora ( )	Até 2 horas ( )		2 a 4 horas ( )			Mais de 4 horas ( )
Avaliação do tempo de hora/aula			Adequado ( )		Pouco ( )			Muito ( )	
Deseja participar de educação permanente			Sim ( )			Não ( )			
**Formulário**									
1. Hemoglobina (g/dL) abaixo da qual a transfusão de concentrado de hemácias está indicada em criança com anemia aguda sem descompensação hemodinâmica:									
a) 6	b) 7	c) 8		d) 9			e) 10		
2. Hemoglobina (g/dL) abaixo da qual a transfusão de concentrado de hemácias está indicada em criança com anemia e choque séptico:									
a) 6	b) 7	c) 8		d) 9			e) 10		
3. Qual o volume (mL/kg) de concentrado de hemácias a ser prescrito em pediatria?									
a) Até 5	b) 5 a 10	c) 10 a 15		d) 15 a 20			e) >20		
4. Qual o tempo (horas) de infusão do concentrado de hemácias em criança com anemia aguda sem sinais de descompensação hemodinâmica?									
a) Aberto	b) Até 1	c) 1 a 4		d) 4 a 8			e) 8 a 12		
5. Em qual das situações está indicado o concentrado de hemácias filtrado?									
a) Anafilaxia prévia à transfusão	b) Insuficiência renal aguda	c) Cardiopatias		d) Doador aparentado			e) Hemoglobinopatias		
6. Em qual das situações está indicado o concentrado de hemácias irradiado?									
a) Imunodeficiência grave	b) Hemoglobinopatias	c) Anafilaxia prévia à transfusão		d) Infecção por dengue			e) Pneumopatias		
7. Em qual das situações está indicado o concentrado de hemácias lavado?									
a) Doador de grupo de risco	b) Insuficiência renal aguda	c) Transplante de medula		d) Anafilaxia prévia à transfusão			e) Imunodeficiência grave		
8. Em qual das situações está indicado o concentrado de hemácias fenotipado?									
a) Hemoglobinopatias	b) Imunodeficiência de IgA	c) Cardiopatias		d) Doador aparentado			e) Recém-nascido		
9. Em qual das situações está indicado o concentrado de hemácias aquecido?									
a) Quimioterapia	b) Transplante de medula	c) Hemoglobinopatias		d) Politrauma e transfusão maciça			e) Anafilaxia prévia à transfusão		
Assinale (X) os sinais e sintomas que se relacionam à reações transfusionais:									
( ) Calafrios									
( ) Aumento de temperatura axilar em 1°C ou mais									
( ) Presença de febre após aumento de 0,5°C									
( ) Alopecia até 72 horas pós-transfusão									
( ) Dor abdominal									
( ) Dor torácica									
( ) Dor no local de infusão da transfusão									
( ) Diarreia									
( ) Hipertensão arterial									
( ) Hipotensão arterial									
( ) Dor de dente									
( ) Dispneia									
( ) Hipóxia									
( ) Icterícia									
( ) Disúria									
( ) Urticária									
( ) Edema localizado ou generalizado									
( ) Otalgia									
( ) Náuseas									
( ) Choque									
( ) Alteração na cor da urina									
( ) Odinofagia
